# Theoretical and Centrifuge Modeling Experimental Monitoring Study on the Seismic Behavior of an Inclined Crack in a Slope

**DOI:** 10.3390/s26134001

**Published:** 2026-06-24

**Authors:** Ning Liang, Yonghua Yu, Zuan Chen, Guodong Yang, Shiyu Li, Yu Zou, Songfeng Guo, Bowen Zheng, Xinyi Guo, Shengwen Qi

**Affiliations:** 1 State Key Laboratory of Lithospheric and Environmental Coevolution, Institute of Geology and Geophysics, Chinese Academy of Sciences, Beijing 100029, China; liangning@mail.iggcas.ac.cn (N.L.); zachen@mail.iggcas.ac.cn (Z.C.); lishiyu@mail.iggcas.ac.cn (S.L.); guosongfeng@mail.iggcas.ac.cn (S.G.); zhengbowen@mail.iggcas.ac.cn (B.Z.); guoxinyi@mail.iggcas.ac.cn (X.G.); qishengwen@mail.iggcas.ac.cn (S.Q.); 2 College of Earth and Planetary Sciences, University of Chinese Academy of Sciences, Beijing 100049, China; 3 China Highway Engineering Consultants Corporation, Beijing 100089, China; yuyonghua@ccccltd.cn (Y.Y.); yangguodong1@ccccltd.cn (G.Y.)

**Keywords:** inclined crack, slope, fracture mechanics, dynamic propagation, seismic behavior, centrifuge shaking table test

## Abstract

Analytical solutions serve as primary benchmarks for verifying model test design, provide rapid predictive tools for preliminary design, and offer fundamental physical understanding of complex structure interaction problems of the geological body. It is essential for ensuring the reliability of experimental results. For the study on slope stability under earthquakes, the seismic behavior of key inclined cracks in the slope is a hot topic, which is a crucial issue in rock mechanics and engineering geomechanics. This paper studies the dynamic propagation of the inclined crack under seismic conditions, proposes the analytical solution of fracture mechanics, and conducts a centrifuge shaking table test accordingly for monitoring and validation. The analytical solution results have been validated experimentally by a centrifuge shaking table test on the seismic behavior of an inclined crack. Results indicate that the amplitude of seismic waves significantly affects crack propagation: the greater the amplitude, the faster the propagation rate. Analysis of crack propagation and maximum surface displacement reveals hysteresis and sudden jumps of surface deformation caused by rock mass structure and locked segments, both in indoor tests and in strong earthquake regions. This paper combines a theoretical and experimental monitoring study, providing a good example of integrating analytical solutions and modeling validation for research on earthquake-induced landslide disasters.

## 1. Introduction

Analytical solutions remain indispensable in geotechnical engineering. They serve as primary benchmarks for verifying model test design, numerical simulation (finite element/discrete element), and back analysis of on-site monitoring, provide rapid predictive tools for preliminary design, and offer fundamental physical understanding of complex structure interaction problems of geology body [1,2,3,4,5]. In complex problems, analytical solutions, although limited by simplified conditions, possess clear physical significance and efficient computation. They are a crucial step in understanding the essence of the problem and guiding test modeling and engineering practice [6,7,8]. Analytical solutions serve as a crucial bridge connecting theory and engineering practice. In today’s era, where experimental simulation is increasingly prevalent, the importance of analytical solutions has not diminished but has instead become an essential prerequisite for ensuring the reliability of experimental results. Without analytical solutions, the validation and interpretation of modeling design would be severely compromised [2,3,9,10,11,12,13].

Slope stability under earthquakes is a crucial issue in rock mechanics and engineering geomechanics. Seismic behavior of the key inclined crack in the slope is well concerned. In the past, scientists and engineers have analyzed slope stability through various methods and theoretically proposed the limit equilibrium method to estimate the safety of homogeneous slopes [14,15]. Some analytical solutions have also been proposed to analyze the collapse mechanism of slopes [16]. For the analysis of the dynamic propagation of cutting cracks vertical to the horizontal plane of slope tops, some scholars have conducted stability analysis using fracture mechanics theory combined with the finite element method [17]. The inclined crack in the slope was studied by Thomas et al. [18] using fracture mechanics to analyze the effect of a linear crack on slope stability, but they only approximately calculated the stress intensity factor at the crack end and measured slope stability by defining a safety factor, without considering time-dependent seismic dynamic response. This paper studied the propagation process of the inclined crack in the slope under seismic conditions, proposed the analytical solution of fracture mechanics, and conducted a validation of the centrifuge shaking table test accordingly.

The centrifuge shaking table test is the most advanced and effective physical simulation method for studying the seismic behavior of crack propagation and instability of slope rock mass structures [19,20]. Its core advantages lie in the authenticity of the stress field, controllability of the process, clarity of mechanism, and reliability of data [21,22]. For the purpose of this paper, which is to conduct experimental verification of analytical solutions, the centrifuge shaking table test can truly reproduce the mechanical behavior and the entire process of crack initiation, propagation, and coalescence of rock mass under seismic waves. The dynamic process is precisely controllable, allowing for the gradual application of various seismic waves for typical instability mechanisms. Compared to field prototype testing, the centrifuge shaking table test is cost-effective and has a shorter cycle; compared to pure numerical simulation, it has strong physical authenticity and direct mechanism revelation [23].

The first section of this paper is an introduction; the second section analyzes the seismic behavior of inclined crack in slope via analytical solution of fracture mechanics; the third section introduces the experimental setup and model preparation on verification by centrifuge shaking table test; the fourth section provides the excitation scheme considering the analytical solution in advance, and the analytical result of fracture mechanics and centrifuge modeling; the fifth section presents the discussion on the result and the hysteresis and sudden jump of surface deformation of slopes both in the centrifuge test and in strong earthquake zones; and finally, a conclusion.

## 2. Fracture Mechanics Analysis of the Seismic Behavior of an Inclined Crack in a Slope

### 2.1. Analysis of Stress Intensity Factors for the Inclined Cracks in the Slope

**Figure 1:**
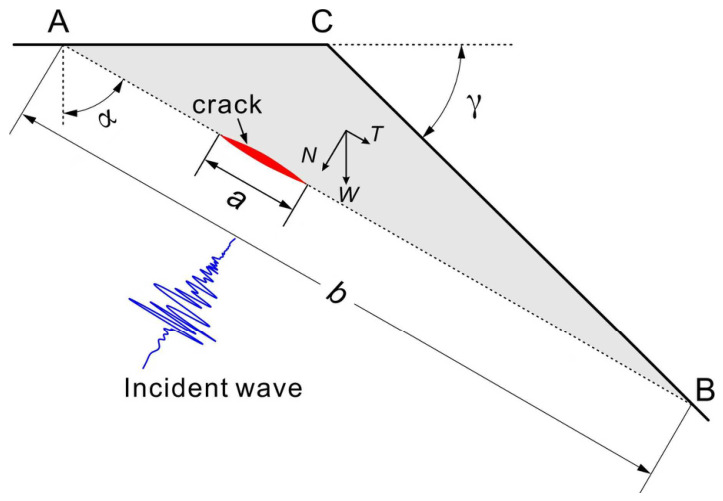
Stress analysis diagram of a slope with an inclined crack.

Figure 1 shows a straight inclined crack hidden inside the slope, with the slope angle γ and the angle α between the inclined crack direction and the vertical direction. The extension lines of both ends of the inclined crack intersect the horizontal plane of the slope top at point A and the slope surface at point B. The length of AB is b. Above the plane AB, the rock mass is subjected to gravity, and the weight is denoted as W.

Figure 1 illustrates that the area of the triangle ABC is as follows:

(1) $$S_{\Delta ABC}=\frac{b^{2}}{2}\left(\sin\alpha \cos\alpha -\cos^{2}\alpha \cot\gamma \right)$$

If the slope is homogeneous and the thickness of the triangle is a unit length, then

(2) $$W=S_{\Delta ABC}\rho$$

where ρ is the density, and

(3) N=Wsinα;T=Wcosα

where W is the weight of rock mass above the plane AB, N is the normal force component perpendicular to the crack, and T is the tangential force component parallel to the crack.

In fracture mechanics [24], the stress intensity factor generated by tensile and shear stress at the tip of the inclined crack is given by

(4) $$K_{Ι}=\sigma \sqrt{\pi a}$$

(5) $$K_{\mathrm{II}}=\left(\tau_{1}+\tau_{2}+F_{T}\right)\sqrt{\pi a}$$

where σ is the stress on the surface of the crack, a is the crack length, $\tau_{1}$ is the frictional force on the inclined crack surface, $\tau_{2}$ is the shear force at a distance from the inclined crack, and $F_{T}$ is the average shear stress generated by seismic waves on the inclined crack surface, as shown in Appendix A.

When considering the effect of seismic waves, the inclined crack surface is generally closed, so the average normal stress $F_{N}$ and average shear stress $F_{T}$ generated by seismic waves on the inclined crack surface are shown in Appendix A.

When $F_{N}+N/b>0$, the frictional force $\tau_{1}$ on the inclined crack surface can be shown as follows:

(6) $$\tau_{1}=-\left[c+\left(F_{N}+\frac{N}{b}\right)\tan\beta \right]$$

where *c* is the cohesive strength and β is the rock mass internal friction angle.

When $F_{N}+N/b<0$, the frictional force $\tau_{1}$ on the inclined crack surface is 0, and there is a tensile stress σ as follows:

(7) $$\sigma =F_{N}+\frac{N}{b}$$

Shear stress $\tau_{2}$ is given by

(8) $$\tau_{2}=\frac{T}{b}$$

### 2.2. Estimation of Dynamic Propagation of Inclined Crack

Theoretically, when the stress intensity factor reaches the fracture toughness, the crack will propagate; when the stress intensity factor is less than the fracture toughness, according to the rheological properties of the material, the crack may begin subcritical propagation.

During crack propagation, if the stress intensity factor remains constant, it means that the crack propagation process is steady-state; if the stress intensity factor continues to increase, crack propagation will enter a non-steady-state process; if the stress intensity factor gradually decreases, crack propagation will slow down and eventually stop. If kinetic and dissipated energy are ignored, crack propagation satisfies the following criteria [25,26,27]:

(9) $$\frac{dU_{c}}{da}+\frac{dSe}{da}=0$$

where $U_{c}$ is the strain energy of crack length a, which is the difference between the elastic strain energy stored in a plate with a crack and the strain energy in a plate without a crack under the same conditions. Se is the surface energy of the crack with the length of a. According to fracture mechanics:

(10) $$\frac{dU_{c}}{da}=-2\left({K_{Ι}}^{2}+{K_{\mathrm{II}}}^{2}\right)\frac{\left(1-\upsilon^{2}\right)}{E}$$

where υ is the Poisson’s ratio.

The change rate in surface energy Se is

(11) $$\frac{dSe}{da}=2\eta$$

where η is the unit surface energy.

Substitute Equations (4)–(8) and (10)–(11) into Equation (9), and as shown in Appendix B, take the derivative of time t to obtain the following equation for the estimation of the dynamic propagation of an inclined crack:

(12) $$\frac{da}{dt}=f\left(t,a\right)$$

## 3. Centrifuge Shaking Table Test Monitoring and Verification

### 3.1. Experimental Setup

**Figure 2:**
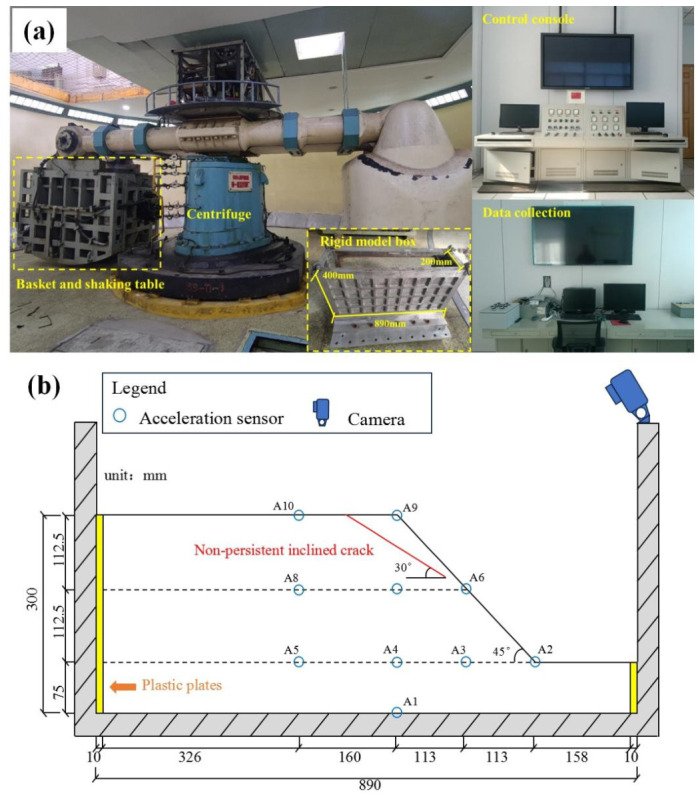
Overview of test equipment and model. (**a**) Centrifuge shaking table at IWHR; (**b**) cross-section of slope model with monitoring (modified from [28]).

The centrifuge shaking table, which belongs to China Institute of Water Resources and Hydropower Research (IWHR), has an effective radius of 5 m and a payload capacity of 1 ton. It is able to be operated at a maximum centrifugal acceleration of 300 g and at a maximum vibration acceleration of 50 g (Figure 2). The model box was built as a solid-walled rigid container 890 mm in length, 200 mm in width, and 400 mm in height.

### 3.2. Model Preparation

The similarity ratio was set to 1:50 (model: prototype) [29,30] and the centrifuge acceleration was set to 50 g. The length, elastic modulus, and density were selected as the main control parameters; see Table 1.

The model was built with a size of 870 mm in length, 200 mm in width, and 300 mm in height, and the slope angle was set to 45°. Based on previous studies [29,30], to fulfill the test requirements, barite powder, quartz sand, gypsum powder, glycerin, and water were selected as similar materials in a specific ratio (0.18:0.71:0.04:0.02:0.05). The gypsum powder had a particle size of 200 mesh, the barite powder was 800 mesh, the quartz sand was in a range of 40–80 mesh, and the glycerin used had a purity of 95%. To mitigate the boundary effect, there were two polystyrene foam plastic plates at the narrow sides of the model box with a thickness of 10 mm. Physical and mechanical parameters of similar material were all obtained by uniaxial compression tests and direct shear tests, which were carried out by a self-developed dynamic direct shear test device [16].

A non-persistent joint, with 190 mm in length, 1 mm in thickness, was preset in the slope model to represent the inclined crack at an angle of 30°. Between the inclined crack and the slope surface, the corresponding length of the rock bridge is 40 mm. Therefore, the length of extension of the inclined crack is 230 mm (Figure 2). The inclined crack was preset as unfilled tensile fractures with zero internal friction and cohesion. To prevent bonding during curing, a polyethylene film was placed at the preset locations of the inclined crack. Finally, the model was hardened indoors for 14 days to stabilize its physical and mechanical properties. This configuration provides a real dynamic propagation of a natural inclined crack [31].

For the seismic behavior record of the slope, a monitoring system was installed (Figure 2). The GoPro Hero 10 Black cameras were fixed from a top view to record and capture high-quality slope activity. The surface displacement of the slope could be analyzed through the non-contact, full-field measurement technique, particle image velocimetry (PIV). PIV uses sequential high-resolution images captured continuously by fixed digital cameras. Prior to testing, either inherent natural speckles or artificially sprayed speckle patterns serve as traceable feature markers on the slope surface. The PIV algorithm conducts cross-correlation analysis within small interrogation windows between adjacent image frames to quantify the spatial offset of matched speckle subsets, yielding the two-dimensional full-field horizontal and vertical displacement distribution of the slope surface. Notably, this technique needs no pre-mounted physical sensors, anchor bolts, or contact transducers on rock and soil masses. Meanwhile, ten unidirectional accelerometers (A1–A10) were embedded in different positions of the slope, and the accelerometer A1 on the shaker base was specifically installed for recording the output wave. The accelerometer is Model B06BM1, following key characteristics with a frequency range of 1–10 kHz (±5% error) and 0.5–11 kHz (±10% error), dynamic range of ±50 g with a resolution of 0.0001 grms, providing a dynamic range of approximately 120 dB, and a natural/mounted resonant frequency of ≥37 kHz.

Recorded from the 1940 Mw 6.9 Imperial Valley earthquake, the El Centro wave is a widely used classic ground motion with the typical waveform and clear spectral characteristics. It features an epicentral distance of 6–12 km, PGA of 0.313 g, PGV of 31.8 cm/s, and a strike-slip focal mechanism. In this study, the El Centro wave was given priority to be selected as the excitation wave, supported by a well-documented dataset of the commonly used strong earthquake record in typical frequency characteristics. Meanwhile, the El Centro wave has been widely used in seismic analysis and dynamic numerical simulation in geotechnical and earthquake engineering, especially shaking table and centrifuge tests [32].

## 4. Result

**Figure 3:**
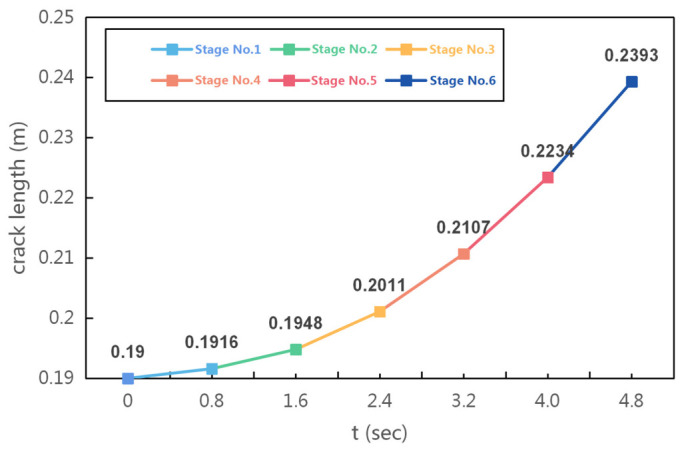
The predicted time history of dynamic propagation of an inclined crack of slope of centrifuge modeling in stages.

**Figure 4:**
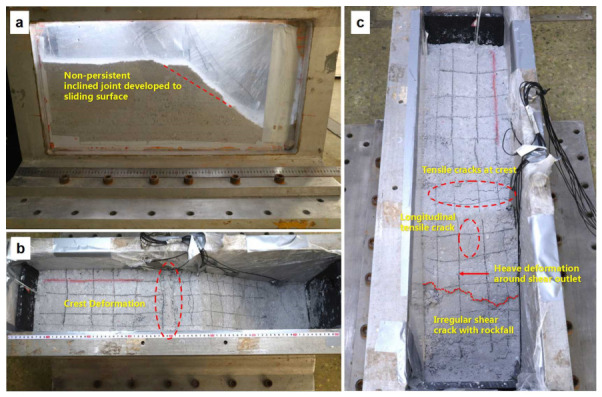
Images of centrifuge shaking table test at the end of stage No. 6. (**a**) Sliding surface; (**b**) crest deformation; (**c**) deformation and failure of slope free surface (modified from [28]).

Before the centrifuge modeling, it is a priority to use analytical solutions of fracture mechanics to repeatedly calculate and compare the dynamic propagation of an inclined crack in a slope under various test designs, which not only can obtain quick answers and preliminary predictions for the tests, but also can establish rigorous verification standards for each key step, greatly ensuring the reliability of the centrifuge shaking table test results. As shown in Table 2 and Table 3, the excitation scheme along with related parameters was decided such that, with increasing amplitudes, six excitation conditions were set up in the centrifuge shaking table test, and the seismic waves, at an interval of 0.8 s, were vertically input from the bottom of the model. All subsequent time-frequency analyses were performed using the prototype-scale seismic signals. The results of the analytical solution by fracture mechanics analysis indicate the predicted dynamic propagation length of the inclined cracks of the slope (see Table 4) and the time history of dynamic propagation of the inclined crack of the slope (see Figure 3) in stages of centrifuge modeling. Accordingly, in Figure 4, images of the centrifuge shaking table test at the end of stage No. 6 indicate that the dynamic propagation of an inclined crack in the slope caused the failure of the slope, accompanied by the recorded development of cracks and deformation of the surface.

## 5. Discussion

The results of an increase in the inclined crack length under seismic waves, both by analytical solution of fracture mechanics and centrifuge modeling, indicate that the amplitude of seismic waves is sensitive to the effect of the inclined crack propagation. The greater the amplitude of the seismic wave, the faster the inclined crack propagation rate; see Table 4.

**Figure 5:**
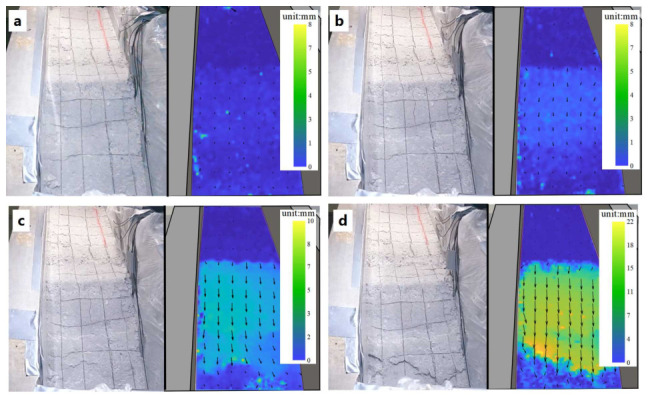
The images and corresponding PIV-derived displacements of slope surface: (**a**) 2.4 s end of stage No. 3; (**b**) 3.2 s end of stage No. 4; (**c**) 4.0 s end of stage No. 5; and (**d**) 4.8 s end of stage No. 6 (modified from [28]).

In most of the stages of centrifuge modeling, as shown in Figure 5, the surface of the slope was recorded by a camera, and the surface deformation analysis of the slope was carried out by PIV. The maximum surface displacements of the slope were 1.5 mm in stage No. 3, 2.3 mm in stage No. 4, 4.2 mm in stage No. 5, and 20 mm in stage No. 6, respectively. If only judging from the images and before stage No. 6, the maximum surface displacements were not obvious, although the max amplitude of seismic waves kept increasing up to 20 g in stage No. 5 of centrifuge modeling.

**Figure 6:**
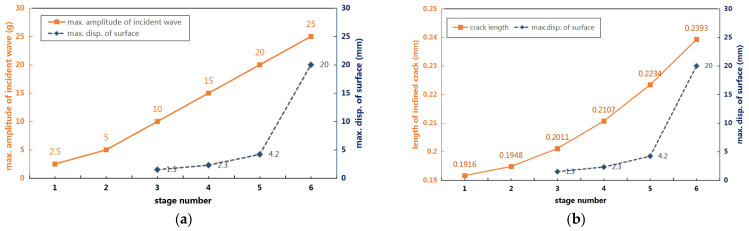
Variations in the maximum amplitude of the incident wave (**a**), crack length (**b**), and maximum surface displacement of the slope in stages of centrifuge modeling.

Meanwhile, further considering Figure 6, the length of the inclined crack in stages is a key factor; it is illustrated that both the acceleration and maximum amplitude of incident wave increase in every stage. Quite the contrary, the maximum surface displacements of the slope in stages only occurred in stage No. 6.

From both theoretical and practical perspectives, the material basis and important internal factors of slope surface deformation (shallow creep, unloading rebound, topple and fall, tensile cracking, local collapse, etc.) are the rock mass structure (structural planes and structural bodies), and the stress/stress field, which must be emphasized, is the main driving and accelerating condition that cannot be neglected. The two factors work together to determine the stability of the slope and the starting position, mode, direction, and scale of slope surface deformation [33,34,35,36,37]. Further, naturally formed by local stress concentration, stress arching, and stress redistribution, the locked segment is a special component unit of rock mass structure mentioned above, being a natural geological body rather than a reinforced structure, which is a typical application and extension of rock mass structure control theory in slope stability research [1,38,39,40,41]. Domestic and foreign scholars define the locked segment as a key structural body/combination that controls rock mass deformation and instability, and the structural integrity and strength of the locked section determine the magnitude and stability of slope surface deformation. The core mechanism is that stress concentration enhances the local rock mass strength, producing locking and anti-deformation and anti-sliding effects [42,43,44,45,46,47]. For this reason, in the paper, taking into account of the inherent rock mass structure of the rock slope, especially the natural locked section under the changing stress field [48,49,50,51,52], the rock mass structure and the locked segment can resist the pressure of deformation and can control and coordinate to mitigate local deformation to the maximum extent, before the sliding surface undergoes overall sliding through progressive failure.

Additionally, it has been shown that the spatiotemporal evolution of the displacement center of the slope surface can effectively determine the landslide stage, identify the locked segment, and further predict the expansion path of the sliding surface [41,43,47]. On this basis, combining Figure 5 and Figure 6, with the increase in seismic inputs in stage No. 3 and No. 4, the almost stable displacement center and region of the slope surface reflect the energy storage, the effect of the rock mass structure, and the formation of the locked segment. In stage No. 5, the relatively larger displacement, which is widely distributed in a similar region without a remarkable displacement center, represents the rock mass structure, and the locked segment continues to be in function without instability. In the final stage, No. 6, dissipation or scattering of the displacement center indicates that the locked segment has been breached and the rock mass structure has become unstable with a sudden jump in displacement of the slope surface. At this moment, some displacement centers gather at the foot of the slope, illustrating that the crack extends to the slope surface and the shear outlet of the slope appears eventually.

**Figure 7:**
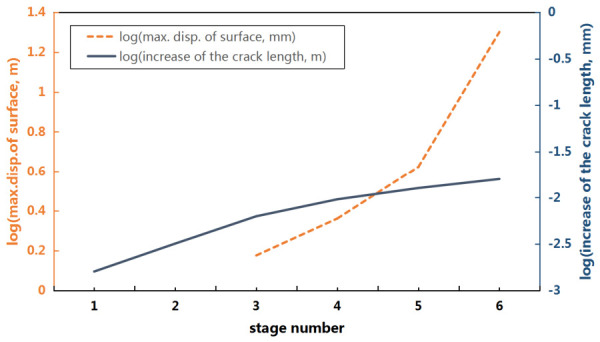
Semi-log plot of variations in the increase in the inclined crack length and maximum surface displacement of the slope in stages of centrifuge modeling.

As shown in Figure 7, further in-depth analysis of the dates obtained by the analytical solution and the centrifuge modeling can effectively echo the discussion above. The semi-log plot indicates very important hidden details that with the significant increase in the maximum amplitude of incident wave ranging from 2.5 g to 25 g in the six stages of centrifuge modeling, the growth rate of the increase in the inclined crack length slowed down significantly after stage No. 3, rather than continuing to increase sharply; correspondingly, when the growth rate of the maximum displacement of surface of slope entered stages No. 4 and No. 5, it increased very little, almost unchanged, and only suddenly increased sharply after entering stage No. 6.

To a large extent, it can be considered that although the exacerbated seismic inputs occur in stages, the rock mass structure and locked segment formed by stress concentration and redistribution in the slope stress field significantly restrain the dynamic propagation of inclined cracks and the deformation of the geological body, affecting the development of slope surface displacement. Only after the stress release and failure of the rock mass structure and locked segment, a large displacement constrained previously became a sudden jump in the surface of the slope.

**Figure 8:**
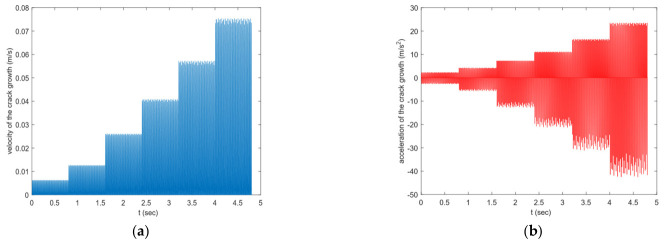
Time histories of velocity (**a**) and acceleration (**b**) of the inclined crack length growth by analytical solution.

In fact, analytical solutions of fracture mechanics can also provide relevant information before the centrifuge modeling. In addition to the predicted time history of dynamic propagation of inclined crack of slope of centrifuge modeling in Figure 3, additional prediction results can be analyzed by derivative operation to obtain the predicted time histories of velocity and acceleration of the inclined crack length growth, respectively (see Figure 8). The lines in the two figures both show a noticeable step-like evolution. Considering the exacerbated seismic inputs over time, the increments of the velocity and acceleration of the inclined crack length growth are difficult to detect between most of the time or stages, as reflected by the bench heights. The obvious suppression of the seismic behavior of the inclined crack propagation can also be attributed to the rock mass structure and locked segment enhanced by the stress field. For this reason, it can be used to infer the potential situation of the lagging deformation and sudden jump of the slope surface.

As a result of the comprehensive discussions on analytical solution and centrifuge modeling above, the statement that the surface deformation of the slope lags and sudden jumps should be more reasonable and appropriate than the statement that the surface deformation of the slope is not obvious in an overly simplistic and misleading manner.

Consistently, in strong earthquake zones, corresponding evidence can also be found to indicate that the hysteresis and sudden jump of surface deformation of slopes occur under and after a series of seismic waves. This type of rock slope is more dangerous and difficult to predict earlier through surface deformation monitoring of space, sky, and ground [53,54,55,56,57,58]. According to studies on the Daguangbao landslide in Gaochuan County, Sichuan, Xinmo landslide in Maoxian, Sichuan, and the landslide in Zelongnong Gully in Tibet, China, it is found that the hysteresis and sudden jump of surface deformation of slopes under earthquakes are based on comprehensive remote sensing interpretation, on-site geological investigation, and seismic records [59,60,61,62,63,64]. This greatly supports the experimental observation and analytical conclusions on the surface deformation of the slope in this paper.

## 6. Limitation

Based on fracture mechanics theory, the analytical solution proposed has predicted and guided the centrifuge shaking table test on the seismic behavior of inclined crack in a slope, but the present theoretical research has certain simplifications compared with real earthquake conditions, and therefore, there are still the following limitations.

The incident seismic waves adopted in this work are simplified to normally incident plane P-waves and SV-waves. In nature, seismic waves have complex incident angles that control slope failure modes like bedding slip and tensile fracture [65,66], and greatly change the acceleration amplification coefficients [67], especially at the slope crest and shoulder [68]. The simplified incident angle of the seismic wave in this study only covers a limited range of cases and cannot fully replicate real-world seismic conditions.

The slope engineering geological model is simplified in this work. It ignores the dynamic response caused by the actual thickness of the slope cover layer, internal differences in the properties of rock masses, and differences in integrity and strength of the same rock masses [69], thus failing to capture the reinforcing effect of geotechnical heterogeneity of the slope on dynamic amplification.

In the centrifuge shaking table test of this study, the test was unable to effectively track and measure the dynamic propagation of the inclined crack in the slope. The process of crack propagation is supported and supplemented by a theoretical analytical solution. The quantitative crack-growth measurements are very important. The solution to this problem will depend on more mature experimental monitoring methods and technologies.

Future research will focus on the coupling relationship between complex incident angles of seismic waves, slope failure modes, and dynamic amplification responses. Meanwhile, detailed geological models that fully reflect the spatial heterogeneity of rock masses will be discussed. Furthermore, more and more field survey and observation data will be necessary to verify and optimize the present theoretical method, making it better adapted to complex in situ seismic environments.

## 7. Conclusions

An analytical solution is important in academic research and engineering practice, serving as the primary benchmark for verifying relevant schemes and a rapid prediction tool for preliminary analysis. The absence of an analytical solution may limit the depth of experimental interpretation and quantitative validation of measurement results. For the mechanical model of the dynamic propagation of the inclined crack in a slope, this paper adapts the method of fracture mechanics to study the seismic behavior of dynamics process of the inclined crack propagation in a slope. The analytical results indicate that the amplitude of seismic waves has a significant effect on the inclined crack propagation.

First, the analytical solution clarifies the relationship between seismic loading and inclined crack propagation. By considering the normal and shear stresses generated by seismic waves on the crack surface, the stress intensity factors and crack-growth process were estimated. The calculated results show that the inclined crack length increases progressively under the input staged El Centro wave. With increasing amplitude of seismic waves, the crack-growth increment, velocity, and acceleration also increase, indicating that the amplitude of seismic waves is a key factor controlling the dynamic propagation of inclined cracks in a slope.

Second, the centrifuge shaking table test reasonably validates the prediction of the analytical solution. Six excitation stages were applied to a slope model with a preset non-persistent inclined crack. The experimental observation results show that the crack gradually propagated toward the slope surface and finally developed into a sliding surface, accompanied by obvious slope deformation and failure in the final excitation stage. This process is consistent with the prediction of the analytical solution.

Third, the monitored slope surface deformation reveals a clear hysteresis and sudden-jump response in the final stage. In the intermediate stages, PIV results show that the maximum surface displacement of the slope remains small, even though the results of the analytical solution indicate continuous crack propagation. A sharp increase in surface displacement of the slope occurs only in the final stage, when the locked segment is breached, and the rock mass structure becomes unstable. Therefore, surface deformation of the slope alone may not fully reflect the internal damage evolution of a cracked slope under earthquake loading. The coupling of analytical crack-growth estimation and surface deformation monitoring provides a more reasonable basis for interpreting the instability of a seismic slope with an inclined crack, showing promising potential for research on seismic landslide prediction.

## Figures and Tables

**Table 1:** Physical parameters of similar material [28].

	Density *ρ* (kg/m^3^)	Elasticity Modulus E (MPa)	Compression Strength $\sigma_{c}$ (MPa)	Poisson’s Ratio *μ*	Cohesion *c* (kPa)	Internal Friction Angle *θ* (°)
Rock	2200.00	59.60	0.44	0.21	72.10	22.10
Joint	/	/	/	/	0	0

**Table 2:** Excitation scheme [28].

Input Waves	Stage Number	Amplitudes (Prototype)	Amplitudes (Model)	Time (Prototype)	Time (Model)
El Centro wave	1	0.05 g	2.5 g	40 s	0.8 s
	2	0.1 g	5 g	40 s	0.8 s
	3	0.2 g	10 g	40 s	0.8 s
	4	0.3 g	15 g	40 s	0.8 s
	5	0.4 g	20 g	40 s	0.8 s
	6	0.5 g	25 g	40 s	0.8 s

**Table 3:** Calculation parameters for the prediction of dynamic propagation of the inclined cracks of the slope in stages of centrifuge modeling.

Parameter Name	Symbol	Unit	Stage Number					
			1	2	3	4	5	6
Initial inclined crack length	a	m	0.19	to be calculated	to be calculated	to be calculated	to be calculated	to be calculated
Angel of inclined crack	α	radian	60°	60°	60°	60°	60°	60°
Length of extension of inclined crack	*b*	m	0.23	0.23	0.23	0.23	0.23	0.23
Slope angle	γ	degree	45°	45°	45°	45°	45°	45°
Friction factor	*tanβ*		0.4061	0.4061	0.4061	0.4061	0.4061	0.4061
Cohesion strength	*c*	MPa	0.0721	0.0721	0.0721	0.0721	0.0721	0.0721
Density	ρ	kg/m^3^	2200	2200	2200	2200	2200	2200
Incident angle of incident wave	*γ*_1_	radian	60°	60°	60°	60°	60°	60°
Velocity of P wave	*c*_1_	m/s	174.63	174.63	174.63	174.63	174.63	174.63
Acceleration of incident wave	*Ag*_0_	g	2.5 g	5 g	10 g	15 g	20 g	25 g
Maximum amplitude of incident wave	*A*_0_	m	1.4718 × 10^−4^	2.9437 × 10^−4^	5.8874 × 10^−4^	8.8311 × 10^−4^	0.0012	0.0015
Incident wave frequency	*f*	Hz	65	65	65	65	65	65
Modulus of elasticity	*E*	MPa	59.6	59.6	59.6	59.6	59.6	59.6
Poisson’s ratio	υ		0.21	0.21	0.21	0.21	0.21	0.21
Surface energy	η	MPam	24	24	24	24	24	24
Duration	t	s	0.8	0.8	0.8	0.8	0.8	0.8

**Table 4:** The predicted dynamic propagation length of the inclined cracks of slope in stages of centrifuge modeling.

Parameter Name	Symbol	Unit	Stage Number					
			1	2	3	4	5	6
Increase in the inclined crack length	*Δ* a	m	0.0016	0.0032	0.0063	0.0096	0.0127	0.0159
Inclined crack length in total	a	m	0.1916	0.1948	0.2011	0.2107	0.2234	0.2393 *

* Length of extension of inclined crack *b* is 0.23 m.

**Figure A1:**
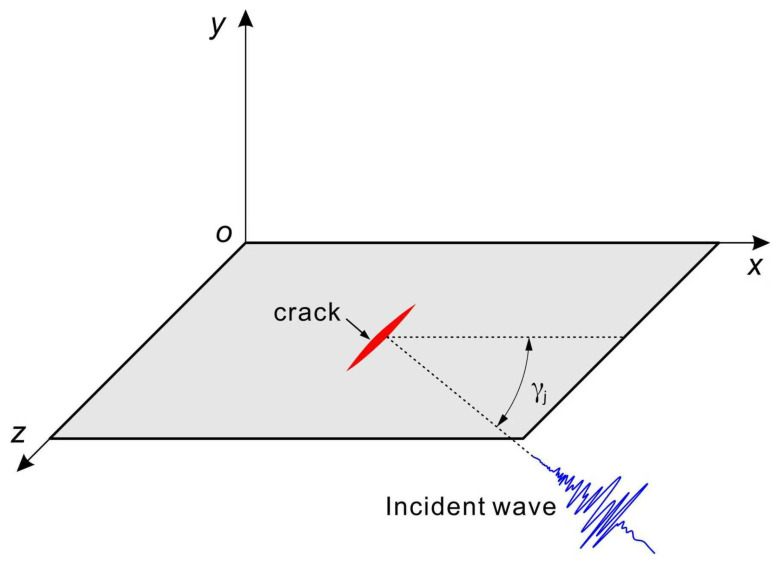
Stress analysis diagram of the incident wave on the crack surface.

## Data Availability

Relevant data are available in this article and from the corresponding author on reasonable request.

## Appendix A. Calculation of Average Stress of Elastic Waves on the Crack Surface

If an incident wave acts on a crack (see Figure A1), the angle between the incident wave and the x-axis is $\gamma_{j}\;(j=1,2)$, corresponding to the P wave, *j* = 1, corresponding to SV wave, *j* = 2. The stress field of the incident P-wave is given by [70]

(A1) $$\sigma_{x}=\sigma_{p}\left(1-2\kappa^{2}\sin^{2}\gamma_{1}\right)\exp\left\{-i\left[\alpha_{1}\left(x\cos\gamma_{1}+y\sin\gamma_{1}\right)+\omega t\right]\right\}\;\sigma_{y}=\sigma_{p}\left(1-2\kappa^{2}\cos^{2}\gamma_{1}\right)\exp\left\{-i\left[\alpha_{1}\left(x\cos\gamma_{1}+y\sin\gamma_{1}\right)+\omega t\right]\right\}\;\sigma_{xy}=\sigma_{p}\kappa^{2}\sin^{2}2\gamma_{1}\exp\left\{-i\left[\alpha_{1}\left(x\cos\gamma_{1}+y\sin\gamma_{1}\right)+\omega t\right]\right\}$$

where

(A2) κ=c2c1=μλ+2μ12=1−2υ2(1−υ)12

where $c_{1}$ and $c_{2}$ are the velocities of the P wave and SV wave, respectively. μ and λ are lame constant, and υ is Poisson’s ratio.

(A3) $$\alpha_{n}=\omega /c_{n}(n=1,2)$$

where *ω* is the circular frequency of the wave.

(A4) $$\sigma_{p}=-\mu \alpha_{1}^{2}\phi_{0}$$

(A5) $$\phi_{0}=A_{0}c_{1}/(\omega \cos\gamma_{1})$$

where $\phi_{0}$ is a constant related to the amplitude of the incident wave. $A_{0}$ is the amplitude of the incident wave.

If the incident wave is an SV wave, the corresponding stress field is given by

(A6) $$\begin{array}{c} \sigma_{x}=-\tau_{sv}\sin2\gamma_{2}\exp\left\{-i\left[\alpha_{2}\left(x\cos\gamma_{2}+y\sin\gamma_{2}\right)+\omega t\right]\right\}\\ \sigma_{y}=-\sigma_{x}\\ \sigma_{xy}=\tau_{sv}\cos2\gamma_{2}\exp\left\{-i\left[\alpha_{2}\left(x\cos\gamma_{2}+y\sin\gamma_{2}\right)+\omega t\right]\right\} \end{array}$$

where

(A7) $$\tau_{sv}=\mu \alpha_{2}^{2}\psi_{0}$$

where $\psi_{0}$ is a constant related to the amplitude of the incident wave, and

(A8) $$\psi_{0}=A_{0}c_{2}/(\omega \cos\gamma_{2})$$

As shown in Figure A1, the normal stress and shear stress generated by elastic waves on the crack surface are $\sigma_{y}$ and $\sigma_{xy}$, respectively. After integrating them along the crack area and dividing them by the length, it is obtained that the average normal stress $F_{N}$ and average shear stress $F_{T}$ on the crack surface.

When the incident waves are P waves

(A9) $$F_{N}=\frac{2\sigma_{p}(1-2\kappa^{2}\cos\gamma_{1})\sin\left(\frac{\alpha_{1}a}{2}\cos\gamma_{1}+\omega t\right)}{a\alpha_{1}\cos\gamma_{1}}\;F_{T}=\frac{2\sigma_{p}\kappa^{2}\sin2\gamma_{1}\sin\left(\frac{\alpha_{1}a}{2}\cos\gamma_{1}+\omega t\right)}{a\alpha_{1}\cos\gamma_{1}}$$

When the incident waves are SV waves

(A10) $$F_{N}=\frac{2\tau_{sv}\sin2\gamma_{2}\sin\left(\frac{\alpha_{2}a}{2}\cos\gamma_{2}+\omega t\right)}{a\alpha_{2}\cos\gamma_{2}}\;F_{T}=\frac{2\tau_{sv}\cos2\gamma_{2}\sin\left(\frac{\alpha_{2}a}{2}\cos\gamma_{2}+\omega t\right)}{a\alpha_{2}\cos\gamma_{2}}$$

## Appendix B. Calculation of the Estimation of the Dynamic Propagation of an Inclined Crack

If the incident wave is a P wave, substitute Equations (4)–(8) and (10)–(11) into Equation (9), and take the derivative of time t to obtain the following equation for the estimation of the dynamic propagation of an inclined crack:

(A11) $$f(a,t)=-f_{un1}/(f_{un2}+f_{un3}-f_{un4})$$

where *a* is the crack length, and

(A12) $$f_{un1}=2\omega \cos(R_{1}a\cos\gamma_{1}/2+\omega t)((f_{n}+NaR_{1}\cos\gamma_{1})f_{n1}+f_{t1}-\tan\beta f_{n1})$$

(A13) $$f_{un2}=2(f_{n}+R_{1}Na\cos\gamma_{1}(f_{n1}f_{c}R_{1}\cos\gamma_{1}/2+R_{1}\cos\gamma_{1}N/b)$$

(A14) $$f_{un3}=2f_{ct}(f_{t1}R_{1}\cos\gamma_{1}f_{c}/2+(-c+T/b-N\tan\beta /b)R_{1}\cos\gamma_{1}-\tan\beta f_{n1}f_{c}R_{1}\cos\gamma_{1})$$

(A15) $$f_{un4}=\eta ER_{1}^{2}(\cos\gamma_{1}{)}^{2}/(\pi (1-\upsilon^{2}))$$

where *ω* is the circular frequency of the wave, $\gamma_{1}$ is the angle between the incident wave and the x-axis, N is the normal force component perpendicular to the crack, *β* is the rock mass internal friction angle, c is the cohesive strength, T is the tangential force component parallel to the crack, b is the length of AB in Figure 1, υ is the Poisson’s ratio, η is the unit surface energy, and

(A16) $$R_{1}=\omega /c_{1}$$

(A17) $$f_{n}=f_{n1}\sin(R_{1}a\cos\gamma_{1}/2+\omega t)$$

(A18) $$f_{n1}=2A(1-2k^{2}\cos\gamma_{1})$$

(A19) $$f_{t1}=2Ak^{2}\sin2\gamma_{1}$$

(A20) $$f_{c}=\cos(R_{1}a\cos\gamma_{1}+\omega t)$$

(A21) $$f_{ct}=f_{t}+(\tau_{1}+\tau_{2})R_{1}a\cos\gamma_{1}$$

where $c_{1}$ is the velocity of the P wave, $\tau_{1}$ is the frictional force on the inclined crack surface, $\tau_{2}$ is the shear force at a distance from the inclined crack, and

(A22) $$A=-ER_{1}^{2}f_{0}$$

(A23) $$k={\left(\frac{1-2\nu }{2(1-\nu )}\right)}^{0.5}$$

(A24) $$f_{0}=\frac{A_{0}c_{1}}{\omega \cos\gamma_{1}}$$

(A25) $$f_{t}=f_{t1}\sin(R_{1}a\cos\gamma_{1}/2+\omega t)$$

where E is the modulus of elasticity, and $A_{0}$ is the maximum amplitude of the incident P wave.

## References

[1] Terzaghi K. (1943). Theoretical Soil Mechanics.

[2] Bishop A.W. (1955). The use of the slip circle in the stability analysis of slopes. Geotechnique.

[3] Mindlin R.D. (1936). Force at a point in the interior of a semi-infinite solid. Physics.

[4] Potts D.M., Zdravković L. (2001). Analytical solutions in geotechnical engineering: A review. Geotechnique.

[5] Sheng D., Sloan S.W., Yu H.S. (2005). The role of analytical solutions in validating numerical models in geomechanics. Int. J. Numer. Anal. Methods Geomech..

[6] Li G.X. (2019). Soil Mechanics.

[7] Venini P., Nascimbene R. (2003). A new fixed-point algorithm for hardening plasticity based on non-linear mixed variational inequalities. Int. J. Numer. Meth. Eng..

[8] Coduto D.P., Yeung M.C.R., Kitch W.A. (2011). Geotechnical Engineering: Principles and Practices.

[9] Terzaghi K. (1962). Stability of steep slopes on hard unweathered rock. Geotechnique.

[10] Poulos H.G., Davis E.H. (1974). Pile Foundation Analysis and Design.

[11] Fairhurst C. (1999). Analytical solutions in geomechanics: Is there a role?. Int. J. Rock Mech. Min. Sci..

[12] Verruijt A., Booker J.R. (1996). Vertical deformation of a homogeneous half-space due to surface loads. Int. J. Numer. Anal. Methods Geomech..

[13] Chen W.F. (1975). Limit Analysis and Soil Plasticity.

[14] Liu S.Y. (2016). Soil Mechanics.

[15] Guo S., Qi S., Yang G., Zhang S., Saroglou C. (2017). An analytical solution for block toppling failure of rock slopes during an earthquake. Appl. Sci..

[16] Qi S., Zheng B., Wu F., Huang X., Guo S., Zhan Z., Zou Y., Barla G. (2020). A new dynamic direct shear testing device on rock joints. Rock Mech. Rock Eng..

[17] Cai W.M., Murti V., Valliappan S. (1990). Slope stability analysis using fracture mechanics approach. Theor. Appl. Fract. Mech..

[18] Thomas M.T., Toddcof D. A field application of fracture mechanics analysis to small crack slope. Proceedings of the 20th USA Symposium on Rock Mechanics.

[19] Adhikary D.P., Dyskin A.V., Jewell R.J. (1997). Centrifuge modelling of toppling failure in rock slopes. Geotechnique.

[20] Kim D., Kim Y., Madabhushi S.P.G. (2011). Centrifuge shaking table tests on seismic response of slopes. Soil Dyn. Earthq. Eng..

[21] Kim Y.-A., Lee H.-I., Ko K.-W., Kwon T.-H. (2022). Centrifuge modeling and analytical validation of seismic amplification in a slope during earthquakes—implications to seismic slope stability analysis. Soil Dyn. Earthq. Eng..

[22] Liu H., Tang A., Zheng T. (2007). Centrifuge modeling of a dry sandy slope response to earthquake loading. Soil Dyn. Earthq. Eng..

[23] Madabhushi S.P.G., Zeng X. Centrifuge shaking table tests on seismic slope failure. Proceedings of the 14th World Conference on Earthquake Engineering.

[24] Yin X.C. (1988). Solid Mechanics.

[25] Chen Z. (2003). Analysis of a microcrack model and constitutive equations for time-dependent dilatancy of rocks. Geophys. J. Int..

[26] Chen Z., Bai W. (2006). Fault creep growth model and its relationship with occurrence of earthquakes. Geophys. J. Int..

[27] Chen Z., Jin Z. (2011). Subcritical dyke propagation in a host rock with temperature-dependent viscoelastic properties. Geophys. J. Int..

[28] Li S.Y., Qi S.W., Zheng B.W., Guo S.F., Luo G.M., Tai D.P., He J.X., Huang X.L. (2026). Seismic behavior of rock slopes containing a partially mobilized sliding surface via centrifuge shaking table testing: Unraveling interaction between seismic load transfer and structural deterioration. Eng. Geol..

[29] He J., Qi S., Zhan Z., Guo S., Li C., Zheng B., Huang X., Zou Y., Yang G., Liang N. (2021). Seismic response characteristics and deformation evolution of the bedding rock slope using a large-scale shaking table. Landslides.

[30] Xu X., Huang Y., Yashima A., Du X. (2022). Failure evolution process of pile-anchor reinforced rock slope based on centrifuge shaking table tests. Eng. Geol..

[31] Tai D., Qi S., Wang T. (2025). Seismic response and progressive failure patterns of shattered slopes controlled by non-persistent joints: Insights from shaking table tests and numerical simulations. Eng. Geol..

[32] Yang C., Fan J., Yue M., Chen G., Wen H., Zhang L. (2025). Progressive failure and critical slowing-down characteristics of bedrock-overburden slope based on shaking table tests. Eng. Geol..

[33] Gu D.Z. (1972). Principles and methods of rock mass engineering geomechanics. Sci. Sin..

[34] Gu D.Z. (1979). Fundamentals of Rock Mass Engineering Geomechanics.

[35] Sun G.Z. (1988). Rock Mass Structure Mechanics.

[36] Wang S.J. (1976). Problems of Engineering Geomechanics of Rock Masses.

[37] Wang S.J. (1994). Engineering Geomechanical Analysis of Slope Stability.

[38] Lajtai E.Z. (1969). Rock bridges in jointed rock masses. Int. J. Rock Mech. Min. Sci. Geomech. Abstr..

[39] Bieniawski Z.T. (1967). Mechanism of brittle fracture of rock: Part II—Experimental studies. Int. J. Rock Mech. Min. Sci..

[40] Einstein H.H., Veneziano D., Baecher G.B. (1983). The effect of discontinuity persistence on rock slope stability. Rock Mech. Rock Eng..

[41] Hoek E., Bray J. (1981). Rock Slope Engineering.

[42] Lajtai E.Z. (1969). Strength of discontinuous rocks in direct shear. Geotechnique.

[43] Einstein H.H., Pariseau W.G. Progressive failure of rock slopes. Proceedings of the 5th International Congress on Rock Mechanics.

[44] Gunzburger Y., Merrien-Soukatchoff V. (2010). Stability analysis of rock slopes with locked segments using a discrete element method. Comput. Geotech..

[45] Zhang Z.Y., Wang S.T., Wang L.S. (1981). Principles of Engineering Geological Analysis.

[46] Qin S.Q., Xue L., Yang B.C., Zhang K. (2018). A physical model predicting instability of rock slopes with locked segments along a potential slip surface. Eng. Geol..

[47] Huang R.Q., Xu Q. (2018). Instability mechanism and physical prediction model of locked-type slopes. Chin. J. Geol..

[48] Xu Q., Li W.L., Dong X.J., Xiao X.X., Fan X.M., Pei X.J. (2017). Preliminary study on characteristics and formation mechanism of Xinmo landslide in Diexi Town, Maoxian County, Sichuan Province. Chin. J. Rock Mech. Eng..

[49] Liu X.J., Zhao C.Y., Li B., Wang W.D., Zhang Q., Gao Y., Chen L.Q., Wang B.H., Hao J.M., Yang X.H. (2025). Identification and dynamic deformation monitoring of active landslides in the Jishishan earthquake area based on InSAR technology. Geomat. Inf. Sci. Wuhan Univ..

[50] Du W.J., Sheng Q., Fu X.D., Hua T., He C., Du Y.X., Zhou Y.Q. (2020). Study on stability and failure mechanism of Yanyangcun landslide under earthquake action. Rock Soil Mech..

[51] Eberhardt E., Stead D., Coggan J.S. (2004). Numerical analysis of initiation and propagation of brittle fracture in a rock slope. Int. J. Rock Mech. Min. Sci..

[52] Luo X., Li L., Zhang Y. (2022). Seismic response and damage model analysis of rocky slopes with weak interlayers. Soil Dyn. Earthq. Eng..

[53] Xu Q., Dong X.J., Fan X.M., Scaringi G., Dai L.X., Li W.L., Zhu X., Pei X.J., Dai K.R., Havenith H.B. (2017). Catastrophic mechanism of the high-speed and long-runout Xinmo landslide in Maoxian, Sichuan. Geol. China.

[54] Yin Y.P., Gao S.H. (2024). Research on high-altitude and long-runout rockslides: Review and prospects. Chin. J. Geol. Hazard Control.

[55] Huang X.H., Li Z.Y., Yu D., Xu Q., Su J.R. (2018). Dynamic process of the June 24, 2017 Xinmo landslide in Maoxian County studied using broadband seismic records. Chin. J. Geophys..

[56] Guo P.Y., Yan X.T., Ji F., Yi L.L. (2023). Physical simulation test of initiation mechanism for Xinmo landslide in Maoxian, Sichuan. J. Eng. Geol..

[57] Fan X.M., Xu Q., Scaringi G., Dai L., Li W., Dong X., Zhu X., Pei X., Dai K., Havenith H.B. (2017). Failure mechanism and kinematics of the deadly June 24th 2017 Xinmo landslide, Maoxian, Sichuan, China. Landslides.

[58] Gao S.H., Yin Y.P., Li B., Gao Y., Wan J.W., Zhang T.T., Gao H.Y. (2024). Dynamic characteristics of the rock-ice avalanche disaster chain in the Zelongnong basin, Yarlung Zangbo river canyon region. J. Eng. Geol..

[59] Huang R.Q., Pei X.J., Cui S.H. (2008). Basic characteristics and formation mechanism of the Daguangbao giant landslide triggered by the Wenchuan earthquake. J. Eng. Geol..

[60] Huang R.Q., Pei X.J., Cui S.H. (2016). Cataclastic characteristics and formation mechanism of rock mass in sliding zone of Daguangbao landslide. Chin. J. Rock Mech. Eng..

[61] Huang R.Q., Pei X.J., Cui S.H. (2015). Study on the catastrophic initiation mechanism of large landslides triggered by the Wenchuan earthquake. Acta Petrol. Sin..

[62] Cui S.H., Pei X.J., Huang R.Q., Zhu L., Meng X.G. (2019). Geological features and causes of the Wenchuan earthquake-triggered large landslides on the right bank of Huangdongzi gully. J. Eng. Geol..

[63] Yin Y.P., Wang W.P., Zhang N., Yan J.K., Wei Y.J., Yang L.W. (2017). Long runout geological disaster initiated by the ridge-top rockslide in a strong earthquake area: A case study of the Xinmo landslide in Maoxian County, Sichuan Province. Geol. China.

[64] Wu H., Li Y.S., Su P.C. (2021). Development characteristics and disaster-causing modes of high-position landslides in the Parlung Zangbo River Basin: A case study of Zelongnong Gully. Geol. Sci. Technol. Inf..

[65] Ren Z., Chen C., Zheng Y., Sun C., Yuan J. (2022). Study on the Influence of Seismic Wave Parameters on the Dynamic Response of Anti-Dip Bedding Rock Slopes under Three-Dimensional Conditions. Sustainability.

[66] Zheng X., Zhao Q., Peng S., Wu L., Dou Y., Chen K. (2024). Analysis of Failure Mechanism of Medium-Steep Bedding Rock Slopes under Seismic Action. Sustainability.

[67] Wang F., Yang Z., Song Z., Liu Y., Tan Y., Liu X. (2024). Influence of SV Wave Oblique Incidence on the Dynamic Response of Arch Dams Under Canyon Contraction. Water.

[68] Jeong S., Moon M., Kim D. (2024). Seismic Wave Amplification Characteristics in Slope Sections of Various Inclined Model Grounds. Appl. Sci..

[69] Zhou Y., Zhao F., Shi Z. (2024). Dynamic Response Mechanism of Bedding Slopes with Alternatively Distributed Soft and Hard Rock Layers Under Different Seismic Excitation Directions: Insights from Numerical Simulations. Materials.

[70] Fan T.U. (2006). Fracture Dynamics: Principle and Application.

